# Occurrence and distribution of antibiotic resistance genes in *Elymus nutans* silage from different altitudes on the Qinghai–Tibetan Plateau

**DOI:** 10.3389/fmicb.2025.1494538

**Published:** 2025-05-19

**Authors:** Xia Zhang, Dongmei Xu, Rina Su, Fuhou Li, Hu Chen, Mengya Jia, Mengyan Chen, Xusheng Guo

**Affiliations:** ^1^School of Life Sciences, Lanzhou University, Lanzhou, China; ^2^Probiotics and Life Health Research Institute, Lanzhou University, Lanzhou, China; ^3^College of Animal Sciences, Shanxi Agricultural University, Taigu, China

**Keywords:** antibiotic resistance, Qinghai–Tibetan Plateau, *Elymus nutans* silage, clinical ARGs, mobile genetic elements

## Abstract

**Introduction:**

Antibiotic resistance genes (ARGs) and antibiotic-resistant bacteria (ARB) have attracted more attentions in fermented feed recently. However, little information is available on the occurrence and distribution of ARGs in ensiled forages in the alpine region of the Qinghai-Tibetan plateau (QTP) with an extremely harsh environment.

**Methods:**

The study investigated the distribution and spread mechanism of ARB and ARGs in *Elymus nutans* silage along 2600 m (low), 3600 m (medium) and 4600 m (high) altitude in the QTP.

**Results:**

The major ARG types in *Elymus nutans* silage were multidrug, aminoglycoside, bacitracin, beta-lactam and polymyxin, while tnpA and IS91 were the dominant mobile genetic elements (MGEs) subtypes in the *Elymus nutans* silage. The dominant ARGs were mainly carried by *Pantoea, Enterobacter, Serratia, and Lelliottia*. Although altitudinal gradient had no influence on the diversity or abundance of other ARGs and MGEs in the *Elymus nutans* silage (*p* > 0.05), the network co-occurrence patterns among ARGs, MGEs, and bacteria in high-altitude silage were more complex than that in low- and medium-altitude silages. The dominant clinical ARGs in the alpine silage were bacA and acrF, and the abundance of clinical ARGs decreased with prolonged fermentation time.

**Discussion:**

This study provides important data on the status of ARGs in ensiled forage from the alpine region of the QTP.

## 1 Introduction

The overuse and misuse of antibiotics have led to the contamination and widespread spread of antibiotic-resistant bacteria (ARB) and antibiotic resistance genes (ARGs). ARGs and ARB have garnered significant attention in livestock feed (Ondon et al., 2021; Zhang Q. et al., 2022). Antibiotic residues can be absorbed by feed crops. Feed crops are made into silage that is used by animals, thereby posing a risk of resistance selection in the animal’s gut flora (Gudda et al., 2020; Sorinolu et al., 2021). Therefore, silage, as a main quality feed for herbivorous livestock, biosafety is closely related to livestock health.

The Qinghai–Tibetan Plateau (QTP), with its high average altitude, cold climate, and low oxygen content, has a negative impact on the development of herbivorous animal husbandry. The natural pasture yield is low and seasonal, and the availability of high-quality feed resources is limited. The feed shortage problem is particularly serious in winter, which makes livestock face the dilemma of insufficient nutrition and consequently makes it difficult to meet their increasing nutritional demands (Ren et al., 2021). *Elymus nutans* (*E. nutans*) is a widely distributed alpine grass on the QTP with high nutritional value and palatability for livestock. It has been reported that *E. nutans* silage can help alleviate the feed supply problem in the QTP regions during the long cold season from November to June (Su et al., 2023). The QTP is a well-developed area for animal husbandry, particularly for breeding yak and Tibetan sheep. In general, the widespread use of antibiotics in animal husbandry induces the production and spread of ARGs. ARGs can enter the environment through animal waste, thereby contaminating soil and vegetation (Sazykin et al., 2021; Shao et al., 2021). Meanwhile, ARGs from wetlands, rivers, and other water resources on the QTP may enter the vegetative growth environment through the water cycle or soil erosion. A study has revealed that ARGs are abundant in the water and sediment of the QTP (Li et al., 2020). Antibiotic resistance in plants can lead to the accumulation of resistance genes in herbivorous livestock products, thereby affecting human health and posing potential health risks (Shao et al., 2021). However, although the presence of ARGs in silage has been found (Wu et al., 2020; Zhang Z. et al., 2022; Zhang et al., 2023), limited information is available on the distribution and transmission mechanisms of ARGs and their risks in ensiled forage from the QTP.

Climatic conditions are among the major factors affecting the biosphere and organisms (Heidari et al., 2020; Shi F. et al., 2023). Temperature and oxygen concentration gradually decline with increasing altitude. Meanwhile, both the soil nutrient and water conditions and the nutrient composition of the vegetation change accordingly (Shi H. et al., 2023). Liu et al. (2022) reported that altitudinal variation affects the succession of microbial communities. A previous study reported that altitude affects the epiphytic microbial structure, nutrient distribution, and fermentation quality of *E. nutans* forage (Su et al., 2023). Yang et al. (2022) confirmed that elevation is a key factor affecting the epiphytic bacterial and fungal communities in *Kobresia pygmaea*. Similarly, Ding et al. (2020) found notable differences in the epiphytic microbial flora among peanut silages in different grasslands. However, the effect of altitude on the distribution characteristics and transmission mechanisms of ARGs in *E. nutans* silage from the QTP remains unclear. Therefore, this study aimed to investigate the response of the distribution and spread of resistant bacteria and ARGs in *E. nutans* silage from the QTP to an altitude gradient. This study is important for understanding the spatial distribution of antibiotic resistance in alpine environments and its influencing factors.

## 2 Methods

### 2.1 *Elymus nutans* silage making

From 13 to 21 August 2021, *E. nutans* plants were collected from three distinct grasslands located in Huangyuan, Xinghai, and Chenduo counties of Qinghai Province. The samples were collected at comparable growth stages from each grassland. In Huangyuan County, the elevations of the three sites were 2,567 m, 2,610 m, and 2,662 m. In Xinghai County, the respective altitudes were 3,555 m, 3,634 m, and 3,647 m. Similarly, in Chenduo County, the sites were situated at altitudes of 4,550 m, 4,582 m, and 4,636 m. We randomly selected each grassland sample from four different locations. The gathered material was promptly sent to the nearby laboratory, where it was then cut into 2 cm pieces with a paper cutter. Once thoroughly mixed, approximately 450 ± 10 g of the cut material was placed into a polyethylene bag (38 cm × 50 cm) and sealed under vacuum. A total of 36 bags were made according to three plots times four replicates times three ensiling periods. These bags were then kept at ambient temperature, approximately 25°C, for 60 days. To assess fermentation traits and conduct metagenomic sequencing, silage samples were extracted at intervals of 7, 14, and 60 days during the ensiling process.

### 2.2 DNA extraction and metagenomic sequencing

DNA was extracted from 20 g of *E. nutans* silage using a Plant Genome DNA Extraction Kit (DP305; TIANGEN, China) according to the manufacturer’s instructions. The extracted DNA was stored at −80°C before analysis. DNA quality was determined using agarose gel electrophoresis (0.5%). DNA concentration was quantified using a NanoDrop 1,000 spectrophotometer (Thermo Scientific, USA).

The metagenomic sequencing library generation was conducted using a DNA Library Prep Kit for Illumina (NEB, USA) following the manufacturer’s specifications (Wu et al., 2020). After library preparation, metagenomic sequencing was performed on an IlluminaSeq 6,000 platform using the 150 paired-end read strategy. For the sample metagenomic datasets, more than 10.0 Gb of reads were sequenced. The metagenomic sequences were filtered to remove low-quality sequences and adapters to obtain clean data (Chen et al., 2018). The clean data were assembled using MEGAHIT software (−-presets meta-large) (Li et al., 2015). The assembled scaffolds were interrupted at the N-junction to obtain sequence fragments called scaftigs (Nielsen et al., 2014). Filtered scaftigs (>500 bp) were used for statistical analysis and gene prediction. Gene prediction was performed as described by Wu et al. (2020).

### 2.3 Annotation and identification of ARGs and mobile genetic elements

The unigenes were compared to the Structured Antibiotic Resistance Genes (SARG) using BLASTN software to search for ARGs (identity >80%, coverage >80%; Yin et al., 2018). Each compared sequence with an identity value greater than the minimum identity value required by the database was assessed to ensure the reliability of resistance gene annotation. The abundance of ARGs in each sample and the resistance mechanism of ARGs were determined after the filtration of comparison results. The ARG and species annotation were conducted based on contigs. Gene coverage was determined using BWA-MEM default cutoff parameters and metagenomic reads mapped to assembled genes (Bogaerts-Márquez et al., 2020).

For MGE annotation, the unigenes obtained from metagenomic sequencing were directly used according to the MGE database (Ma et al., 2013). The unigenes were annotated as plasmid-like, integron-like, transposase-like, insertion sequence transposase (ist)-like, or insertion sequence (IS)-like genes if the threshold similarity for gene annotation was >90%and the length of the unigenes matched the reference sequence by at least 25 amino acids.

### 2.4 Host identification analysis

After quality control, the contigs were spliced using MEGAHIT (Li et al., 2015). The open reading frame (ORF) was identified using Prodigal (Hyatt et al., 2010), and then the ORFs were made on redundant using CD-HIT to obtain a non-redundant gene set (Fu et al., 2012). The abundance of each gene was calculated using Salmon software (Patro et al., 2017). The non-redundant gene set was compared to the SARG and MGE databases using BLASTX for ARG and MGE annotation, respectively, and then the annotated ARG and MGE contigs were further compared to the NCBI-NR database for taxonomic classification. The data processing and graph drawing were completed using the “dplyr” package and “ggplot 2” package (Wickham et al., 2020), respectively.

### 2.5 Identification of clinical ARGs

To identify clinical resistance genes in *E. nutans* silage, a BLAST search was conducted between the ARG reference sequence in the SARG database and the Pathosystems Resource Integration Center (PATRIC) database (Snyder et al., 2007). The threshold of comparison was that the consistency and matching length were >80%. The final matched SARG reference sequence was the clinical ARGs. Subsequently, the reference sequence-level gene abundance obtained using the ARG-OAP tool was matched with the identified clinical ARGs to determine the gene abundance of clinical ARGs in the samples (Yin et al., 2018).

### 2.6 Data analysis

Tukey’s test was used to compare the means of ARG types, ARG hosts, and MGE types in R statistical software, and the means were considered significantly different when the *p-*value was <0.05. The total ARG abundance in each group was visualized using box plots. Principal coordinate analysis (PCoA) was carried out using the “ape” package. The “linkET” package was used to perform a Mantel analysis of the relationship between fermentation quality and ARGs, MGEs, and bacteria (Huang et al., 2021). All additional charts not specifically mentioned were created using the “ggplot 2” package of the R software. Network analysis was performed using BH calibration and Spearman’s correlation. Genes or species with discovery rates <60% were eliminated before correlation analysis, and genes or species with low abundance (gene abundance >5 ppm and bacterial species abundance >0.5%) were removed to lower the number of false-positive results. Gephi version 0.9.6 was used to create network diagrams, which were reserved for results with correlation coefficients >0.8 and *p*-values <0.5.

## 3 Results

### 3.1 Effects of altitude on ARG distribution and resistance mechanisms in *Elymus nutans* silage

**Figure 1:**
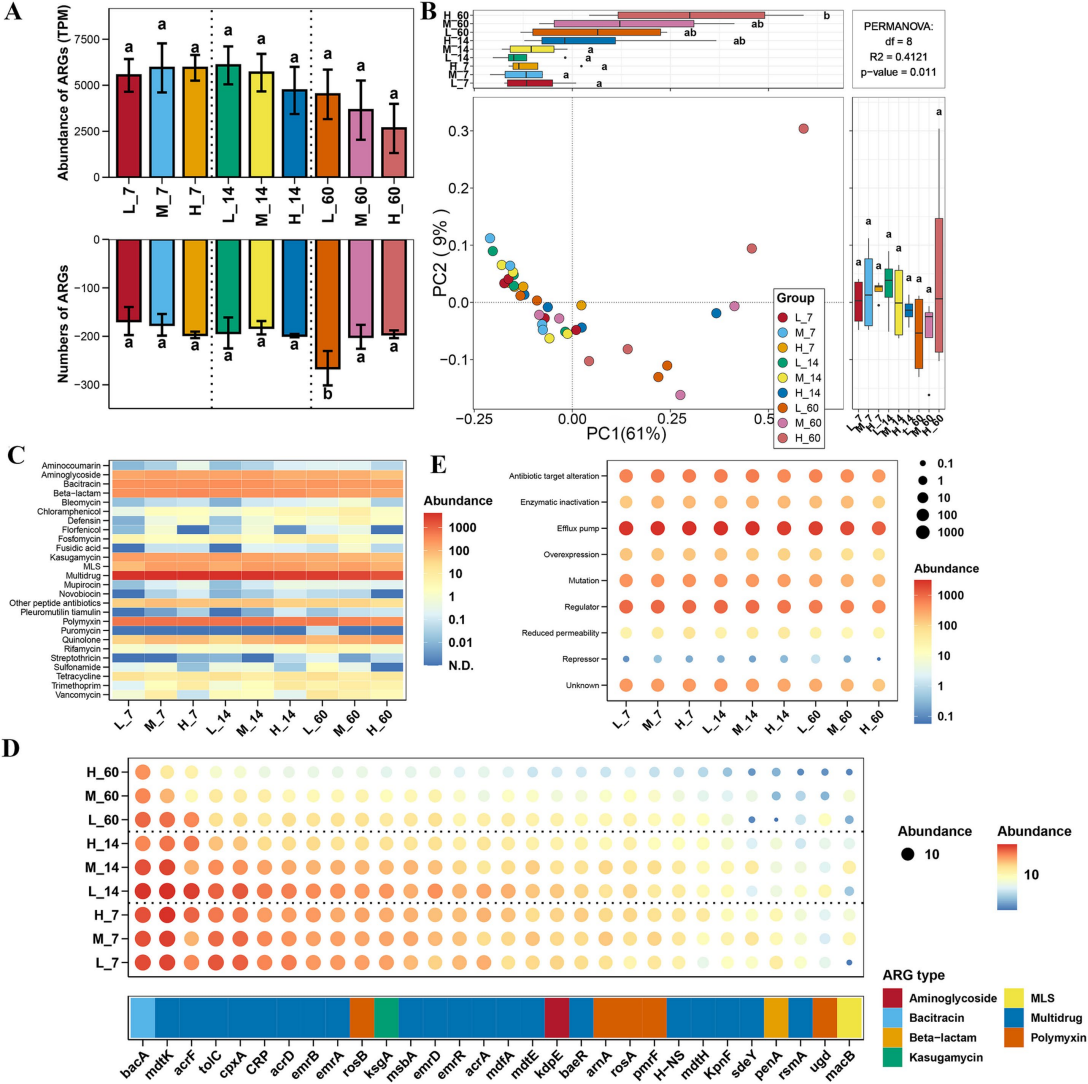
Variation in the diversity and abundance of antibiotic resistance genes (ARGs) in *Elymus nutans* silage at different altitudes. **(A)** Total number and abundance of ARGs; **(B)** principal coordinate analysis (PCoA) of ARGs. **(C)** Abundance of ARG type; **(D)** abundance of ARG subtype; and **(E)** abundance of ARG resistance mechanism. L, low altitude; M, middle altitude; H, high altitude; 7, 14, and 60 represent 7, 14, and 60 days of fermentation, respectively.

The diversity and total abundance of ARGs in *E. nutans* silage were not significantly affected by altitude (*p* > 0.05) (Figures 1A,B). After 60 days of ensiling, the number of ARGs at low altitudes was higher than that at medium and high altitudes (*p* < 0.05) (Figure 1A). The results showed that multiple drugs, aminoglycosides, bacitracin, beta-lactams, and polymyxins were the major ARG types in *E. nutans* silage (Figure 1C). Similarly, the abundance of ARG types in *E. nutans* silage was not affected by altitude (*p* > 0.05). After 60 days of ensiling, the abundance of streptothricin was higher at low altitudes than at medium and high altitudes (*p* < 0.05). The results of the ARG subtypes showed that *bacA*, *mdtK*, *acrF*, *tolC*, *cpxA*, *CRP*, and *acrD* were the major ARG subtypes in *E. nutans* silage (Figure 1D). At the same altitude, the effect of fermentation time on ARG was negligible. The efflux pump was the major ARG resistance mechanism in each group, followed by regulation and antibiotic target alteration (Figure 1E). Altitude and fermentation time had no major effects on ARG resistance in *E. nutans* silage.

### 3.2 Effects of altitudes on changes in microbial communities in *Elymus nutans* silage

**Figure 2:**
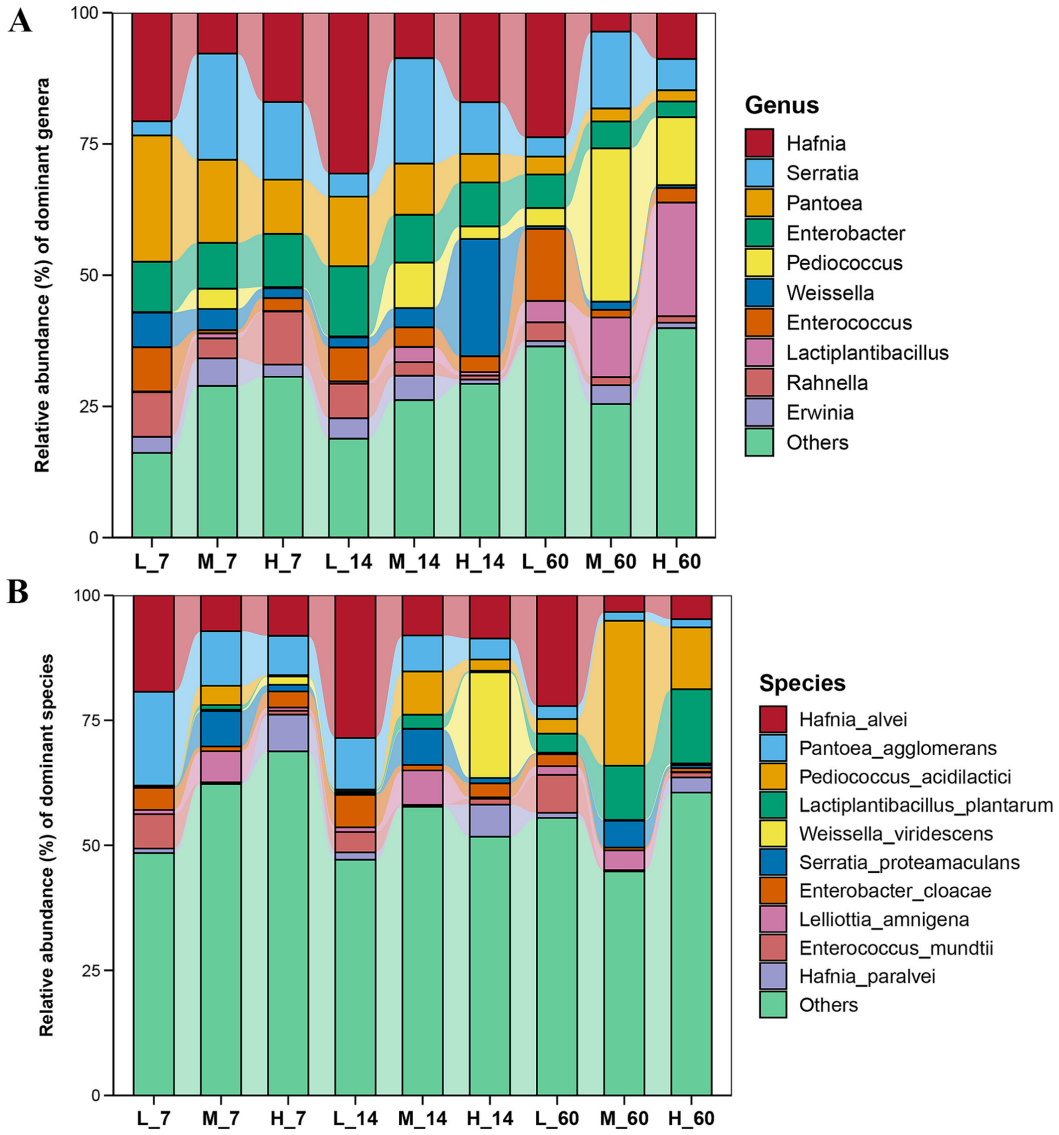
Succession of bacterial communities in *Elymus nutans* silage at different altitudes. **(A)** Relative abundance of dominant bacteria at the genus level; **(B)** Relative abundance of dominant bacteria at the species level. L, low altitude; M, middle altitude; H, high altitude; 7, 14, and 60 represent 7, 14, and 60 days of fermentation, respectively.

During ensiling, the abundance of *Pantoea* and *Pantoea agglomerans* gradually decreased with increasing altitude (*p* < 0.05) (Figure 2). The abundances of *Serratia*, *Serratia proteamaculans*, *Pediococcus*, and *Pediococcus acidilactici* were the highest at middle altitudes (*p* < 0.05). The abundances of *Hafnia* and *Hafnia alvei* were the highest at low altitudes (*p* < 0.05). At all three altitudes, the abundances of *Lactiplantibacillus* and *Lactiplantibacillus plantarum* increased with increasing fermentation time.

### 3.3 Effects of different altitudes on ARG hosts in *Elymus nutans* silage

**Figure 3:**
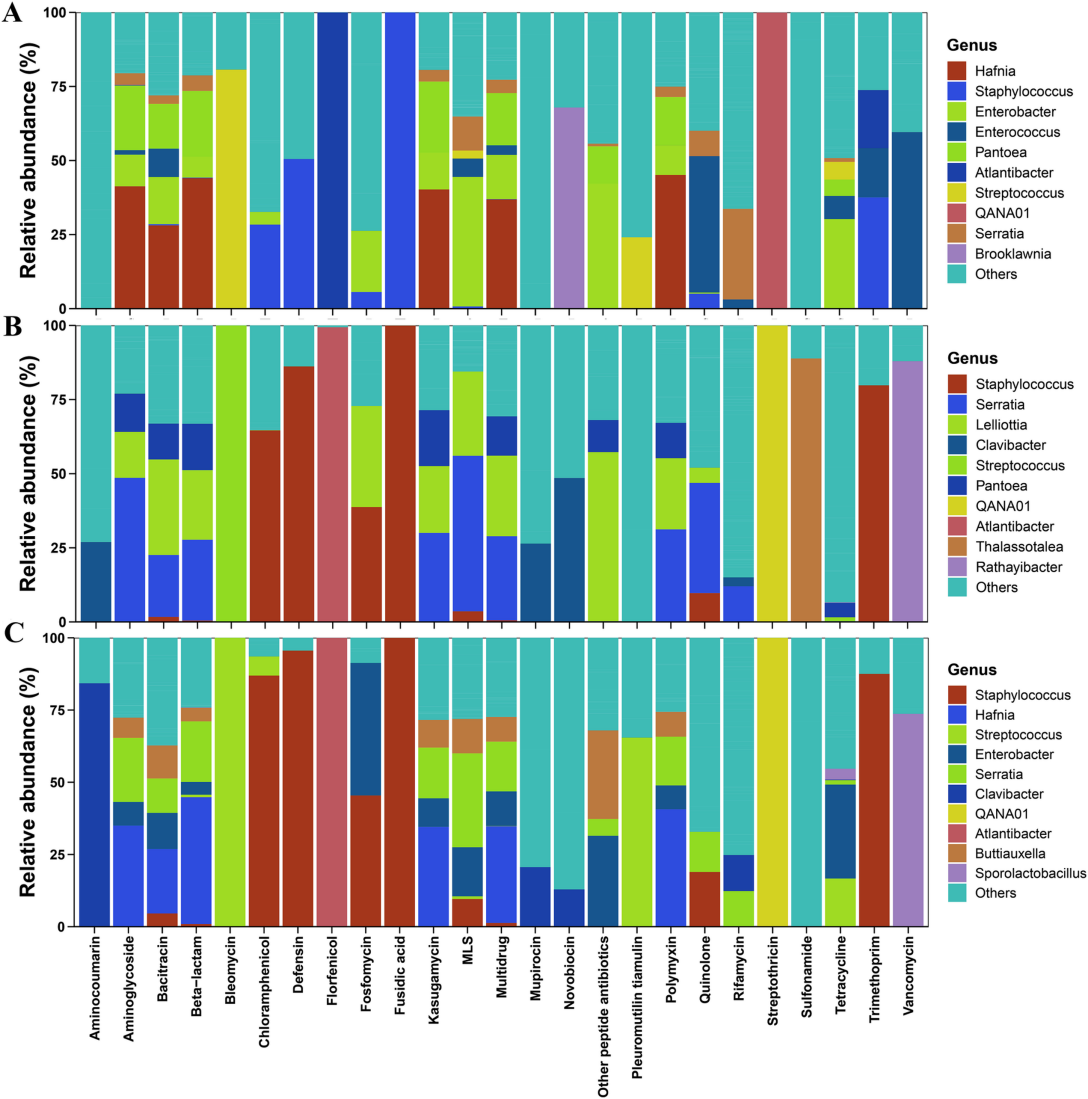
Antibiotic resistance gene (ARG) hosts information in *Elymus nutans* silage at different altitudes. **(A)** Low altitude; **(B)** middle altitude; and **(C)** high altitude.

At low altitudes, *Hafnia*, *Pantoea*, and *Enterobacter* were the major carriers of some dominant ARGs (multidrug, aminoglycoside, bacitracin, beta-lactam, and polymyxin) in *Elymus nutans* silage (Figure 3A). At mid-altitudes, *Serratia*, *Lelliottia*, and *Pantoea* were the primary carriers of the dominant ARGs (multidrug, aminoglycoside, bacitracin, beta-lactam, and polymyxin) in *Elymus nutans* silage (Figure 3B). At high altitudes, *Hafnia*, *Serratia*, *Enterobacter*, and *Atlantibacter* were the main carriers of the dominant ARGs in *Elymus nutans* silage (Figure 3C). In addition, *QANA01* was the only carrier of streptothricin at all three altitudes. *Atlantibacter* was the only carrier of florfenicol. *Staphylococcus* was the only carrier of fusidic acid.

### 3.4 Effects of altitude on the distribution and carriers of MGEs in *Elymus nutans* silage

**Figure 4:**
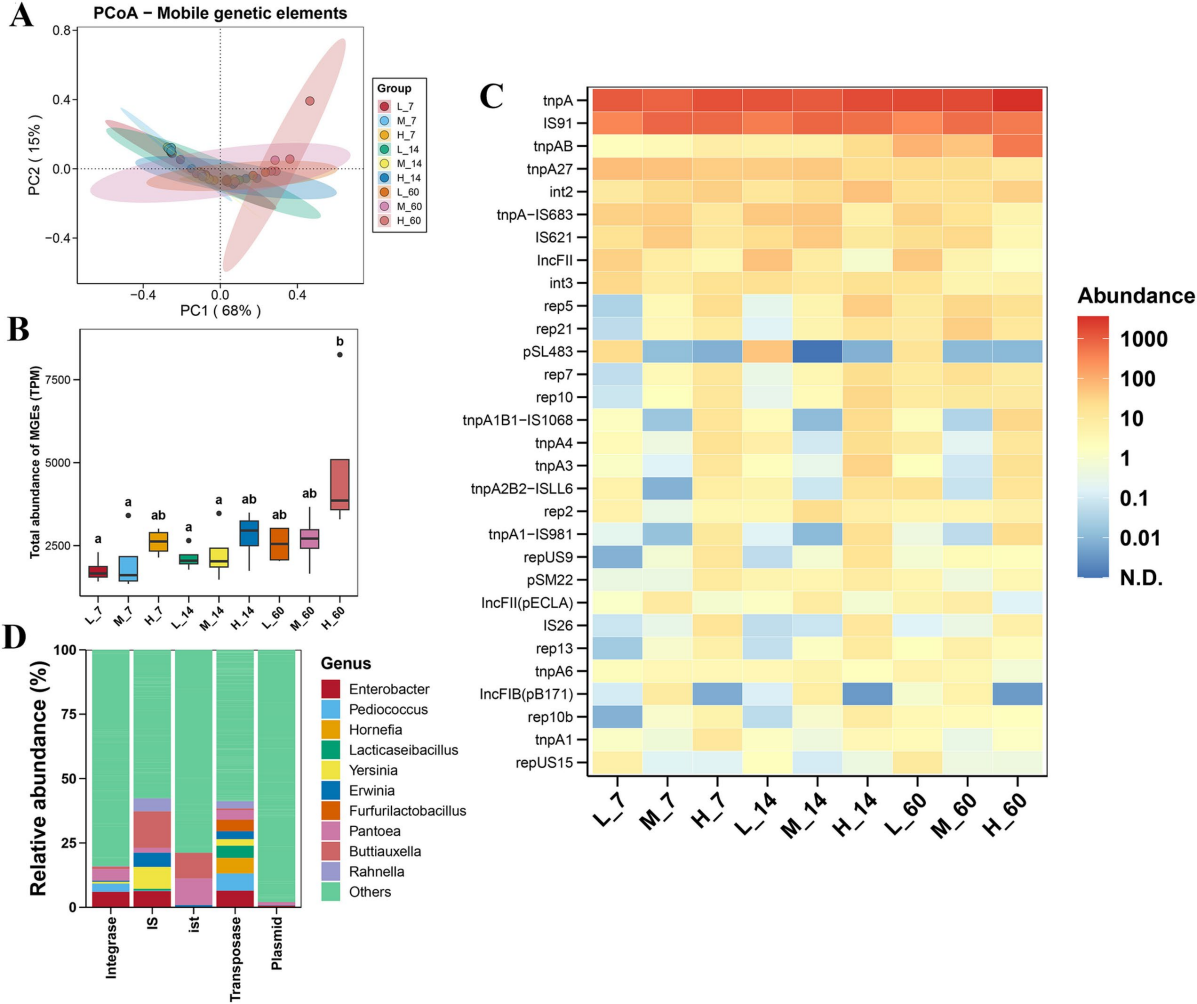
Variation in the diversity and abundance of mobile genetic elements (MGEs) in *Elymus nutans* silage at different altitudes. **(A)** Principal coordinate analysis (PCoA) of MGEs; **(B)** total abundance of MGEs; and **(C)** composition and abundance of top 30 MGE subtypes. **(D)** Hosts of MGE types (including IS, integrase, plasmid, and transposase). L, low altitude; M, middle altitude; H, high altitude; 7, 14, and 60 represent 7, 14, and 60 days of fermentation, respectively.

As shown in Figure 4A, the MGE-based PCoA showed no remarkable separation among the different altitudes. During ensiling, altitude had no effect on the total abundance of MGEs, except that at 60 days of fermentation, the total abundance of MGEs at high altitudes was higher than that at low and medium altitudes (*p* < 0.05) (Figure 4B). The *tnpA* and *IS91* were the dominant MGE subtypes in each group (Figure 4C). After ensiling for 7 days, the abundances of *IS26* and *tnpA1* increased at high altitudes compared to low and medium altitudes (*p* < 0.05). After 60 days of ensiling, the abundances of *tnpAB* and *tnpA1-IS981* were higher at high altitudes than at low and medium altitudes (*p* < 0.05). *Pantoea* was the main carrier of the plasmid (Figure 4D). *Enterobacter, Pediococcus, Hornefia*, and *Lacticaseibacillus* were the major transposase carriers. The dominant hosts of *ist* were *Pantoea* and *Buttiauxella*. The dominant IS hosts were *Buttiauxella, Yersinia, Enterobacter, Erwinia*, and *Rahnella*. *Enterobacter*, *Pantoea*, and *Pediococcus* were the main carriers of integrases.

### 3.5 Correlation among MGEs, ARGs, and bacterial communities

**Figure 5:**
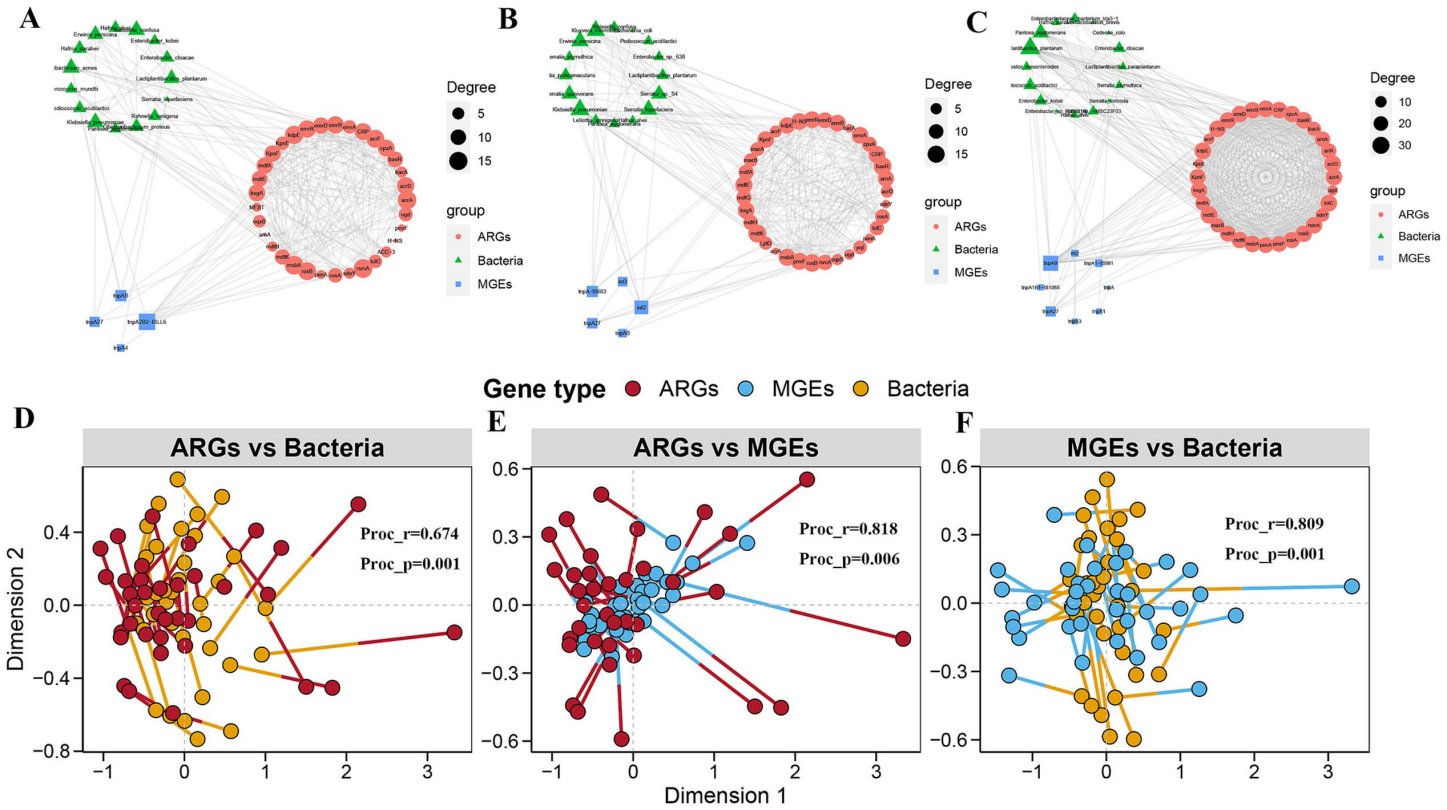
Network co-occurrence pattern of bacteria with antibiotic resistance genes (ARGs) and mobile genetic elements (MGEs) in *Elymus nutans* silage at low, middle, and high altitudes, respectively **(A–C)**. Procrute analysis of bacteria with ARGs and MGEs in *Elymus nutans* silage with low, middle, and high altitudes, respectively **(D–F)**.

At low altitudes, 33 ARGs and 4 MGEs highly co-occurred with 15 bacteria (Figure 5A). Among these visual associations, *tnpA2B2-ISLL6* was highly correlated with the majority of the target ARGs. *Weissella confusa*, *Bacterium acnes*, and *Erwinia persicina* were the major bacteria associated with ARGs. These results suggest that *tnpA2B2-ISLL6* may be the major mediator that promotes ARG transmission in *E. nutans* silage at low altitudes. At medium altitudes, 36 ARGs and 5 MGEs showed high co-occurrence with 16 bacteria (Figure 5B). *Erwinia persicna*, *Serratia liquefaciens*, and *Klebsiella pneumoniae* were the major bacteria associated with ARGs. *tnpA-IS683*, *int2*, and *tnpA27* were MGEs highly correlated with most target ARGs. At high altitudes, 32 ARGs and 8 MGEs highly co-occurred with 16 bacteria (Figure 5C). *Lactiplantibacillus plantarum*, *Pantoea agglomerans*, and *Enterobacter cloacae* were the chief bacteria associated with ARGs. *tnpAB* was an MGE that correlated with the majority of the target ARGs. Notably, the coexistence patterns of ARGs, MGEs, and bacterial species at high altitudes were more complex than those at low and medium altitudes, and more ARG subtypes were present at high altitudes. This suggests that high-altitude conditions may increase the risk of horizontal gene transfer (HGT) of ARGs in *E. nutans* silage. Procrustes analysis further showed a close correlation among the three (Figures 5D–F).

### 3.6 Influences of fermentation quality on ARGs, MGEs, and microbes in *E. nutans* silage

**Figure 6:**
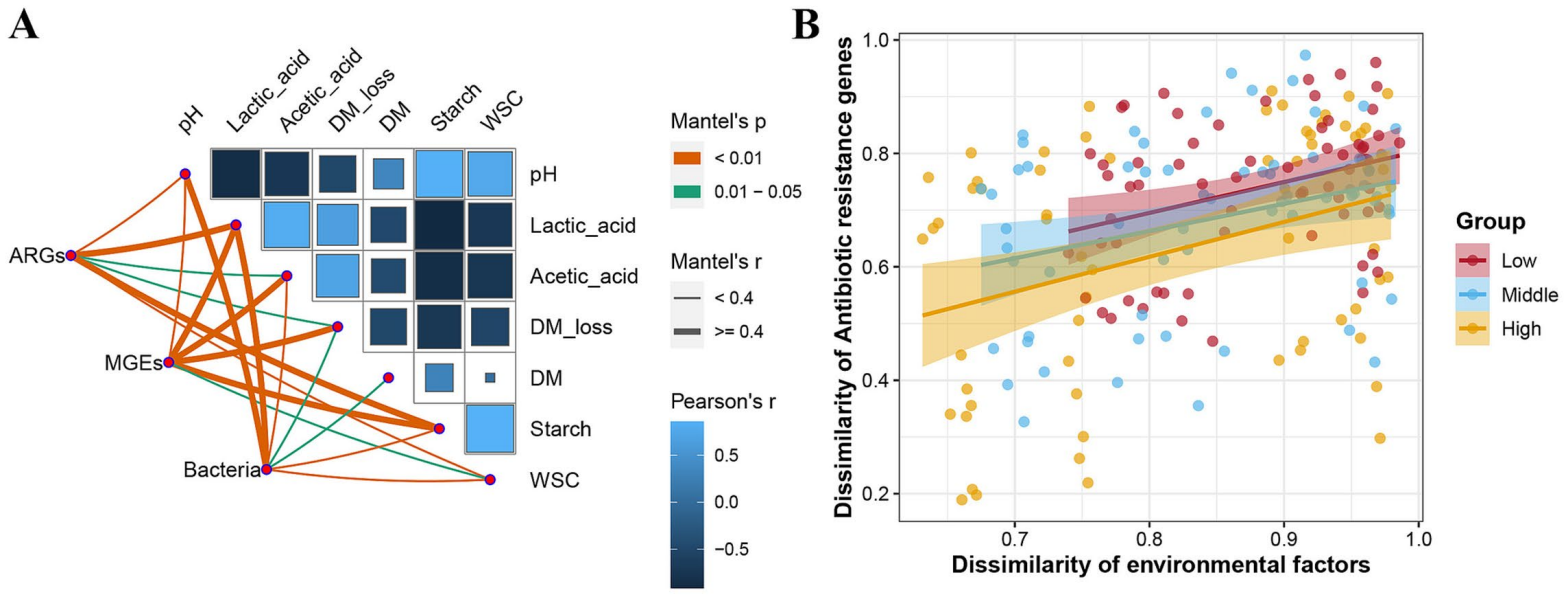
Influences of fermentation quality on bacteria, antibiotic resistance genes (ARGs), and mobile genetic elements (MGEs) in *Elymus nutans* silage. **(A)** Mantel analysis shows the correlation between fermentation quality and ARGs, MGEs, and bacterial community. **(B)** Distance-dependent curve of dissimilarity between ARGs and fermentation quality. The closer the slope of the fitted curve is to one, the weaker the distance-dependent similarity between environmental factors and ARGs. DM, dry matter; WSC, water-soluble carbohydrates.

Data on the fermentation quality have been presented in a previously published paper (Su et al., 2023). The correlation between fermentation characteristics, ARGs, MGEs, and microbial composition was analyzed using the Mantel test (Figure 6A). Lactate and starch levels were highly correlated with ARG levels (r > 0.4, *p* < 0.01). Changes in MGEs were highly correlated with lactate, acetate, DM loss, and starch content (r > 0.4, *p* < 0.01). Bacterial communities were associated with the majority of the fermentation indicators, with pH and lactate levels as the key factors. Figure 6B further shows that there was a positive correlation between the dissimilarity of ARGs and the dissimilarity of environmental factors. The correlation between them increased with the increase in altitude.

### 3.7 Clinical ARGs in *E. nutans* silage from different altitudes

**Figure 7:**
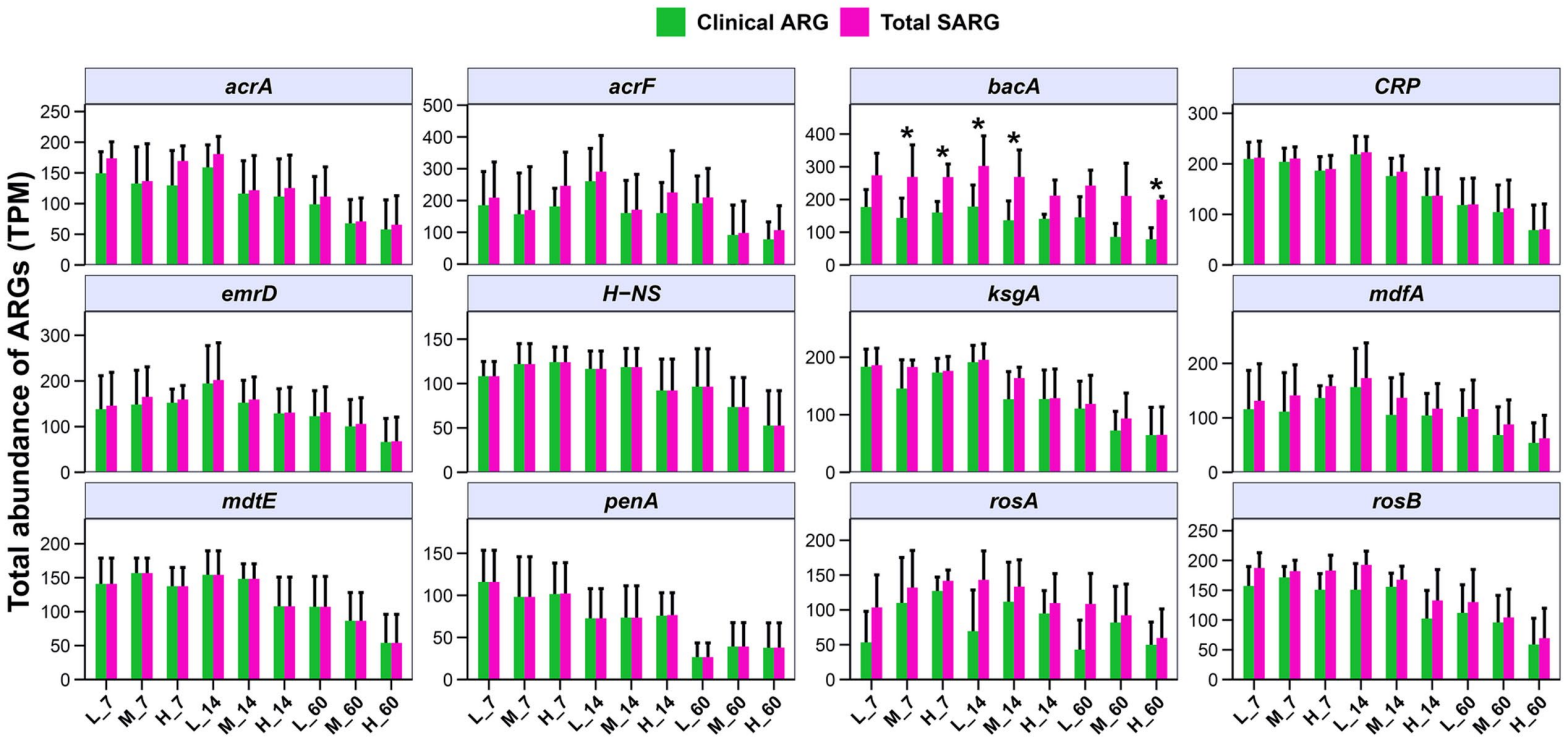
Abundances of the top 12 clinical antibiotic resistance genes (ARGs) in *Elymus nutans* silage at different altitudes. L, low altitude; M, middle altitude; H, high altitude; 7, 14, and 60 represent 7, 14, and 60 days of fermentation, respectively.

Some ARGs in human pathogens have been obtained from environmental bacteria through HGT. Therefore, evaluating the clinical ARGs carried by pathogens in *E. nutans* silage at different altitudes is important. In the present study, the top 12 clinical ARGs in *E. nutans* silage collected at different altitudes were screened (Figure 7). The top 12 clinical ARGs included one beta-lactam (*penA*), one bacitracin (*bacA*), one kasugamycin (*ksgA*), two polymyxins (*rosA* and *rosB*), and seven multidrug genes (*acrA*, *acrF*, *CRP*, *emrD*, *H-NS*, *mdfA*, and *mdtE*) in *E. nutans* silage collected at different altitudes. The *bacA* and *acrF* were the predominant clinical ARGs in each group. In addition, the abundance of clinical ARGs was decreased with prolonged fermentation time, in particular, the abundances of *acrA*, *CRP*, *ksgA*, *penA*, and *rosB*. Notably, the majority of clinical ARGs (*acrA*, *acrF*, *CRP*, *emrD*, *H-NS*, *mdfA*, *mdtE*, and *penA*) with 90% amino acid homology in *E. nutans* silage have high sequence similarity to ARGs in human pathogens. This indicates that ARGs in *E. nutans* silage from different altitudes on the QTP pose a concern, with at least some potentially entering the clinical domain.

## 4 Discussion

Key ARGs in *E. nutans* silage from the QTP include those conferring resistance to multidrug, aminoglycoside, bacitracin, beta-lactam, and polymyxin antibiotics, which are commonly used in both animals and humans. The reasons for the presence of ARGs in *E. nutans* silage from the QTP include environmental factors and anthropogenic activities, according to Wang N. et al. (2024). The QTP is a developed area for animal husbandry, particularly the breeding of Tibetan livestock breeds (Zhou et al., 2018). The widespread use of antibiotics in animal performance may induce ARG production in animals, which may then enter the environment through animal waste, thereby contaminating the soil and vegetation (Muhammad et al., 2020; Zhang Q. et al., 2022). In addition, although the agricultural activity on the QTP is relatively low, any agricultural or other economic activities may involve the use of antibiotics, thereby indirectly contributing to the spread of ARGs in the environment. The present study showed that altitude did not affect the abundance of ARG type in *E. nutans* silage. Yang et al. (2019) also found no correlation between altitude and ARG abundance. Although altitude has an important effect on the ecological environment, it is not a unique factor in determining the abundance of anoxia or ARGs on the QTP (He et al., 2016; Wang S. et al., 2024). A previous study has found that many environmental factors, such as air temperature, vegetation coverage, soil, and precipitation, can affect ARG abundance (Shi F. et al., 2023; Miao et al., 2023). Therefore, the direct effect of altitude on ARG abundance may be weakened by a combination of multiple factors. In addition, we found that fermentation time had less effect on the ARG of *E. nutans* silage at the same altitude. This result contributes to the combined action of the anaerobic environment, microbial community, silage quality, raw materials, and ensiling conditions (Yang et al., 2019). Finally, the abundance of streptothricin in low-altitude silage was higher than that in middle- and high-altitude silages. This could be because the soil microbial communities at low altitudes are richer and more diverse due to the relatively warm and humid climate. These microbes may carry streptothricin, which is passed on to *E. nutans* through horizontal gene transfer.

Different altitudes have different environmental factors (such as climate and soil properties), vegetation types, biological interactions, and human disturbances. These factors affect the succession of microbial communities (Xie et al., 2023). The current study also found that changes in altitude caused changes in certain microbes (*Pantoea*, *Serratia*, *Pediococcus*, and *Hafnia*). ARG host analysis showed that the carriers of the dominant ARG differed at the three altitudes. The carriage and distribution characteristics of ARG at different altitudes may be determined by environmental factors, microbial communities, genetic elements, and anthropogenic activities (Qian et al., 2021). In addition, it was found that the ARG carriers in *E. nutans* silage on the QTP were mainly harmful bacteria (*Hafnia*, *Pantoea*, *Enterobacter*, *Serratia*, *Lelliottia*, and *Atlantibacter*). A previous study found that ARG carriers in alfalfa silage are also harmful epigenetic bacteria (Zhang et al., 2023). Mobile genetic elements play an important role in the HGT of ARG by capturing, integrating, and transferring them. The current study found that altitude has no significant effect on the total MGE abundance. However, the abundances of *tnpAB*, *TNPA1-IS981*, *IS26*, and *tnpA1* increased in high-altitude silage. The *tnpA* and *IS91* were the dominant MGE subtypes in each group, and the major hosts of the different MGE subtypes were different. These results may be attributed to the fact that different MGEs carry different genetic codes that determine their host type (Frost et al., 2005).

The major carriers (*Pantoea*, *Enterobacter*, and *Pediococcus*) of MGEs and ARGs were identical. This indicated that MGEs and ARGs formed close symbiotic and co-evolutionary relationships during the evolutionary process (Bengtsson-palme et al., 2018; Zhou et al., 2021). Procrute analysis showed that the correlation between the number of MGEs and ARGs was relatively strong compared to that between microbes and the number of ARGs. It was inferred that the influence of microbes on ARGs was obtained through HGT mediated by MGEs (Duan et al., 2019). Notably, the network concurrence patterns of ARGs, MGEs, and bacteria in high-altitude silage were more complex than those in the low- and medium-altitude silages. This further suggested that high-altitude conditions increased the risk of ARG transmission in *E. nutans* silage.

Environmental factors play an important role in the ARG distribution. It was considered that environmental factors directly affect the survival of microorganisms and indirectly change the distribution of ARGs (Wang et al., 2020; Delgado-Baquerizo et al., 2022; Li et al., 2023). Zhang et al. (2024) reported that the dry matter content and fermentation property indirectly affected the changes in ARGs in whole-plant corn silage. Therefore, determining the factors influencing ARGs in *E. nutans* silage was helpful for controlling resistance propagation. The present results showed that the fermentation quality (DM loss, pH, lactic acid, and acetic acid) affected ARGs, MGEs, and microbes in *E. nutans* silage, especially lactate and starch contents. In summary, the occurrence and spread of ARGs in *E. nutans* silage were affected by several factors. Notably, ARGs in ecological environments may be transmitted to animals or humans through the food chain and pose a potential threat to public health. This study found that the ensiling process reduced the abundance of clinical ARGs, especially acrA, CRP, ksgA, penA, and rosB. The majority of the clinical ARGs (acrA, acrF, CRP, emrD, H-NS, mdfA, mdtE, and penA) in *E. nutans* silage had a high sequence similarity to ARGs found in human pathogens. This suggests that at least some of these ARGs have possibly entered the clinical domain, posing potential risks to public health and medical treatment. Therefore, it is important to strengthen the monitoring and management of ARGs related to human health in *E. nutans* silage. This can help prevent and control the spread of antibiotic resistance. In addition, it is necessary to further explore the possibility, transmission mechanism, and potential risks of ARGs from silage transfer to humans through the food chain in future studies.

## 5 Conclusion

The results of this study indicate that multidrug, aminoglycoside, bacitracin, beta-lactam, and polymyxin are the major ARG types in *E. nutans* silage from the alpine region of the QTP. The dominant MGE subtypes in *E. nutans* silage were tnpA and IS91, and the efflux pump was the major resistance mechanism against ARGs. *Pantoea*, *Enterobacter*, *Serratia*, and *Lelliottia* were the primary carriers of the dominant ARGs in *E. nutans* silage. Although altitude and ensiling time had no effect on the abundance of the majority of ARGs and MGEs in *E. nutans* silage, the abundance of streptothricin was higher in low-altitude silage than in middle- and high-altitude silages. Moreover, the abundances of *tnpAB* and *tnpA1-IS981* were higher in high-altitude silage than in low- and middle-altitude silages. Network co-occurrence patterns of ARGs, MGEs, and bacteria in high-altitude silage were more complex than those in low- and medium-altitude silages. Clinical ARGs were also found in *E. nutans* silage, but their abundance was decreased with prolonged fermentation time, particularly the abundances of *acrA*, *CRP*, *ksgA*, *penA*, and *rosB*.

## Data Availability

The original contributions presented in the study are included in the article/supplementary material, further inquiries can be directed to the corresponding author.
